# Nasopharyngolaryngoscopy as a Triage Tool for Airway Compromise in Angioedema: A Retrospective Cohort Study

**DOI:** 10.7759/cureus.23759

**Published:** 2022-04-02

**Authors:** Shameek Gayen, Tejas Sinha, Veena Dronamraju, Bilal Lashari, Huaqing Zhao, Santosh Dhungana

**Affiliations:** 1 Pulmonary and Critical Care Medicine, Temple University Hospital, Philadelphia, USA; 2 Health Sciences, Temple University, Philadelphia, USA

**Keywords:** intubation, medical triage, nasopharyngolaryngoscopy, mild respiratory distress, angioedema

## Abstract

Background

Airway compromise and respiratory failure are feared complications of angioedema leading to intensive care unit (ICU) admission. However, few of these patients decompensate. There is a paucity of tools that predict airway compromise in patients with angioedema, and it is unclear if automatic triage to the ICU is warranted. We analyzed patients admitted to our tertiary center ICU with angioedema for “airway watch” to find a way to triage those at greatest risk of respiratory decompensation.

Methods

We performed a retrospective review of patients with angioedema admitted to our ICU between 2017 and 2020. Data collected included demographics, comorbidities, nasopharyngolaryngoscopy (NPL) findings, need for intubation, and length of stay. Descriptive analysis and subsequent ANOVA or T-test statistical analysis was performed to determine the relationships between individual variables and outcomes. Categorical variables were compared using Pearson's Chi-squared test or Fisher's exact test where applicable. Continuous variables were compared using a Mann-Whitney U test.

Results

Of 134 patients admitted to our ICU, 63 (47%) required intubation, primarily in the emergency department (92.1%). Of those who required intubation, 61.9% had abnormal NPL findings in contrast to 25.35% of patients who did not require intubation (p<0.0001). Normal NPL findings had a negative predictive value for requiring intubation of 86.5%. Abnormal NPL findings had a positive predictive value for requiring intubation of 68.4%.

Conclusion

While airway compromise is a serious complication of angioedema, there is scant evidence to support triage to the ICU for those not intubated immediately. The majority of patients with angioedema who required intubation had abnormal NPL findings, and the majority of those with normal NPL findings did not require intubation. This suggests that NPL findings in patients with angioedema can help with triage to the ICU.

## Introduction

Angioedema can cause upper airway compromise; as such, patients with angioedema are frequently admitted to the intensive care unit (ICU) for the initial phase of their inpatient care. Angioedema is either histamine-related and allergic in nature, or bradykinin-related, which is more severe and longer acting. Medications are the most commonly implicated cause of bradykinin-mediated angioedema [1]. Angiotensin-converting enzyme inhibitors (ACEi) are the classical causative agent and the most common, followed by non-steroidal anti-inflammatory drugs [2].

The available evidence suggests that airway compromise requiring intubation is an uncommon complication of angioedema. Several studies have shown an intubation rate for airway compromise in angioedema patients ranging from 7% to 11% [3,4]. In Sandefur et al.’s study, they noted that 70% of all presenting angioedema patients were discharged from the emergency department (ED) [4]. However, the risk of respiratory collapse and difficulty obtaining an airway in an emergent setting both contribute to providers recommending observation of these patients in an ICU, colloquially termed an “airway watch”.

It is suggested that otorhinolaryngology (ENT) consultation and evaluation to determine the severity and location of edema can assist in triaging angioedema patients with risk of upper airway compromise. Ishoo et al. recommend a staging scheme for the severity of illness in angioedema based on symptoms, signs, and fiberoptic laryngoscopic findings in eighty patients. The authors reported a 7 percent risk of intubation in patients with lingual edema (stage III), which increased to 24 percent with the presence of laryngeal edema (stage IV). The odds of ICU admission correlated with the severity of the edema [5]. Similar findings are shared by Bentsianov and colleagues in a multicenter retrospective review, indicating that the most accurate assessment of disease severity involves evaluating symptoms in conjunction with laryngoscopic examination [6]. Guidelines from the French Reference Centre for Angioedema (CREAK) published in a consensus statement recommend against evaluating with anatomical markers alone; however, they do not recommend for or against routine nasopharyngolaryngoscopy (NPL) in determining the disposition of patients with angioedema [7]. The consensus statement put forth by Moellman et al. recommends laryngoscopy in all patients with head and neck angioedema with any lingual involvement or upper airway complaints, but with level of evidence C, as those with airway involvement may require airway intervention or ICU monitoring [8].

There are also other investigations that are considered possible tools for airway permeability assessment in the ED, such as laryngeal sonography, which could clearly visualize the extent of edema [9]. Despite these suggestions, there is a paucity of validated, comprehensive tools to predict who may benefit from closer monitoring. We performed a retrospective review of patients admitted to our intensive care units with angioedema to determine the association between abnormal NPL findings and subsequent intubation and to develop a simplified triage tool for ICU observation versus general floor observation.

## Materials and methods

We performed a retrospective chart review of patients admitted to the ICU at Temple University Hospital for angioedema between the years 2017 and 2020. Patients were identified via ICD-10 diagnosis code T78.3 (angioneurotic edema). Patients with angioedema who were not admitted to the ICU were excluded. The policy of our institution's emergency department is that any patients with angioedema admitted to the hospital be triaged to the ICU. We collected the following data: demographics (including age, gender, race, body mass index), pre-existing lung disease and obstructive sleep apnea (OSA), provision of flexible nasopharyngolarygoscopy (NPL), findings on NPL (normal or abnormal), need for intubation, length of ICU stay, and length of hospital stay. NPL findings were categorized as abnormal if any of the following structures seen on NPL had edema identified by the operator: nasopharynx, Eustachian tube orifices, post-pharyngeal wall, vallecula, epiglottis, pyriform sinuses, base of tongue, false vocal cords, and true vocal fold movement. NPL was performed by ENT or ED residents supervised by their respective attendings. To our knowledge, there were no quality controls in place to determine the accuracy of findings aside from the structures defined.

The primary outcome measured was the difference in NPL findings between intubated patients and non-intubated patients. Secondary outcomes included a comparison of ICU days and total length of stay between intubated and non-intubated patients. Descriptive analysis and subsequent ANOVA or T-test statistical analysis was performed to determine the relationships between individual variables and outcomes. Categorical variables were compared using Pearson's chi-squared test or Fisher's exact test where applicable. Continuous variables were compared using a Mann-Whitney U test. A p-value of ≤0.05 was considered statistically significant. Data analysis was performed using SPSS Statistic Software version 25 (IBM Inc., Armonk, New York).

This study was conducted in accordance with the amended Declaration of Helsinki, and was approved by the Western Institutional.

## Results

We identified 134 patients with angioedema who were admitted to our institution’s ICU between 2017 and 2020. Of these patients, 63 (47%) required intubation, and 71 (53%) did not require intubation. Comparison of demographic information, including age, sex, race/ethnicity, and BMI, and the respiratory comorbidities of lung disease and OSA between intubated and non-intubated patients showed no significant difference (Table 1). A significantly higher number of patients who required intubation had respiratory distress or stridor (p<0.0001) and an oxygen requirement (p<0.0001) than patients who did not require intubation (Table 2).

**Table 1:** Patient demographics and comorbidities BMI: body mass index, OSA: obstructive sleep apnea, SD: standard deviation

	Overall (n=134)	Intubation		
		Yes (n=63)	No (n=71)	p-value
Mean age in years (SD)	59 (44.3-73.7)	60 (47.1-72.9)	58 (41.7-74.3)	0.539
Sex				0.603
Male (%)	60 (44.8)	30 (47.62%)	30 (42.25%)	
Female (%)	74 (55.2)	33 (52.38%)	41 (57.75%)	
Race				0.551
White	19 (14.18%)	8 (12.70%)	11 (15.49%)	
Black	83 (61.94%)	43 (68.25%)	40 (56.34%)	
Asian	1 (0.75%)	0 (0.00%)	1 (1.41%)	
Other	27 (20.15%)	11 (17.46%)	16 (22.54%)	
Unknown	4 (2.99%)	1 (1.59%)	3 (4.23%)	
BMI in kg/m2 (SD)	31.47 (7.22)	32.46 (7.45)	30.59 (6.94)	0.136
Lung disease	48 (35.82%)	22 (34.92%)	26 (36.62%)	0.859
OSA	18 (13.43%)	9 (14.29%)	9 (12.68%)	0.805

**Table 2:** Respiratory clinical characteristics of patients with angioedema

	Overall (n=134)	Intubation		
		Yes (n=63)	No (n=71)	p-value
Respiratory distress or stridor on presentation	32 (23.88%)	29 (46.03%)	3 (4.23%)	< .0001
Required oxygen on presentation	64 (47.76%)	50 (79.37%)	14 (19.72%)	< .0001

Of the 134 patients, 94 (70.1%) received NPL and 40 (29.9%) did not receive NPL (Table 3). There was no standardized timing of NPL beyond being performed in the emergency department. Of the 63 patients who required intubation, 58 (92.1%) were intubated upon presentation in the emergency department. A higher proportion of patients requiring intubation had abnormal NPL findings than patients not requiring intubation, while a higher proportion of patients not requiring intubation had normal NPL findings. Statistical analysis showed that the difference in NPL findings between intubated and non-intubated patients was significant (p<0.0001). Detailed abnormal NPL findings are shown in Table 3, with nasopharyngeal, epiglottic, and true vocal cord edema seen more in patients requiring intubation. We found 37 patients with normal NPL findings; only five of these patients (13.5%) required intubation, giving normal NPL findings a negative predictive value for requiring intubation of 86.5%. We found 57 patients with abnormal NPL findings; 39 of these patients (68.4%) required intubation, giving abnormal NPL findings a positive predictive value for requiring intubation of 68.4%.

**Table 3:** NPL findings among angioedema patients *Some patients had more than one abnormal NPL finding. Abnormal NPL findings broken down by specific structure involvement (nasopharynx, Eustachian tube orifice, post-pharyngeal wall, vallecula, epiglottis, pyriform sinuses, base of tongue, false vocal cords, true vocal cords). NPL: Nasopharyngolaryngoscopy

	Overall (n=134)	Intubation		
		Yes (n=63)	No (n=71)	p-value
NPL performed	94 (70.15%)	44 (69.84%)	50 (70.42%)	1.000
NPL findings				< .0001
Normal NPL findings	37 (27.61%)	5 (7.94%)	32 (45.07%)	
Abnormal NPL findings*	57 (42.54%)	39 (61.90%)	18 (25.35%)	
Nasopharynx, n (%)	11 (8.2%)	8 (12.7%)	3 (4.2%)	
Eustachian tube orifice, n (%)	0 (0%)	0 (0%)	0 (0%)	
Post-pharyngeal wall, n (%)	8 (6.0%)	5 (7.9%)	3 (4.2%)	
Vallecular, n (%)	1 (0.7%)	0 (0%)	1 (1.4%)	
Epiglottis, n (%)	16 (11.9%)	10 (15.9%)	6 (8.5%)	
Pyriform sinuses, n (%)	1 (0.7%)	0 (0%)	1 (1.4%)	
Base of tongue, n (%)	9 (6.7%)	5 (7.9%)	4 (5.6%)	
False vocal cords, n (%)	5 (3.7%)	2 (3.2%)	3 (4.2%)	
True vocal cords, n (%)	16 (11.9%)	15 (23.8%)	1 (1.4%)	
NPL not performed	40 (29.85%)	19 (30.16%)	21 (29.58%)	

The number of ICU days on average was 3.4 days in intubated patients and 1.08 days in non-intubated patients (p=0.0005). The total length of stay, on average, was 6.63 days in intubated patients and 2.65 days in non-intubated patients (p<0.0001) (Table 4).

**Table 4:** ICU and total length of stay among angioedema patients ICU: Intensive Care Unit

	Overall (n=134)	Intubation		
		Yes (n=63)	No (n=71)	p-value
ICU days (n)	2.17 (3.61)	3.4 (4.98)	1.08 (0.46)	< .0001
Total hospital days (n)	4.52 (5.15)	6.63 (6.52)	2.65 (2.26)	< .0001

## Discussion

The existing evidence shows that the development of airway compromise requiring intubation is uncommon amongst patients with angioedema. However, intervention is necessary if airway collapse does occur, and in a subset of individuals, it may be beneficial to observe respiratory status in a controlled environment. Predicting which patient is most at risk for the development of complications is an arduous task. The lack of standardized tools and insensitivity of physical exams in predicting glottic and laryngeal edema are challenges to providing patients with the optimal disposition [6]. In clinical practice at our institution, there appears to be a trend toward increased intensive care utilization to observe for the development of airway obstruction. However, only a minority of patients develop airway threats significant enough to warrant intubation and mechanical ventilation. It is important to note that those with a prior history of allergic reactions, chronic allergic rhinitis, or asthma may have a higher risk of developing severe angioedema with airway collapse. As such, triage for angioedema is optimized with an interdisciplinary team including ED, ENT, critical care, and allergy specialists [10]. The development of standardized triage tools could lead to cost-effective intensive care utilization.

Of ICU admissions at our institution for angioedema, we identified an intubation rate of 47%. This proportion is higher than current published literature, although it is important to note that our study only examined patients admitted to the ICU. Studies that utilized ED cohorts are likely to have lower rates of patients requiring intubation [3,4]. Prior studies suggested that risk for intubation correlated with the anatomical location and severity of edema on NPL. Our findings suggest that an abnormal NPL correlates well with the risk of intubation, while a normal NPL correlates well with not requiring intubation. Normal NPL findings had a very high negative predictive value for the risk of intubation in patients with angioedema, which suggests that normal NPL findings can be a successful screening tool for low risk of intubation and need for ICU admission in this population. While we found that certain areas, such as nasopharynx, epiglottis, and true vocal cords, were more affected in patients requiring intubation, the significance of these findings is limited by the small sample size. As such, we suggest that the simplified interpretation of normal versus abnormal NPL can act as a triage tool for ICU admission and monitoring versus general floor admission in this patient population.

We suggest performing an NPL as early as possible for any concerning clinical presentation. This may help guide the triaging physician to the optimal disposition of the patient. Additionally, urgent ENT consultation is not always readily available, and there is often hesitancy to utilize risk stratification procedures based on advanced anatomy. A simplified triage tool based on the presence of normal or abnormal NPL findings may allow for increased adoption of NPL amongst non-ENT providers, including emergency room physicians and intensivists, although we realize that there could be a significant proportion of non-ENT providers that may not be comfortable performing NPL.

Conversely, care of ICU patients may be de-escalated earlier in the presence of normal NPL findings. Although only focusing on patients with angioedema admitted to the ICU, it is apparent from the available data that the risk of intubation in the presence of a normal NPL is minimal, and these patients can be safely observed in a non-critical care setting.

Limitations

A major limitation of our study is that the patient population studied includes only those already triaged in the ICU. As such, our study does not include patients with angioedema seen and discharged from or observed in the ED; this group could be a significant and clinically relevant portion of the angioedema patient population. By virtue of including only ICU admitted patients, our study is not representative of all patients who present with acute angioedema, and there may have been airway emergency events that were missed from the analysis. Additionally, the majority of our patients were intubated upon presentation to the ED, which could bias both patient selection and the NPL operator if NPL was performed after intubation. There was no standardized timing for performing NPL, though this likely did not affect ICU admission decision making, given our institution's policy for patients with angioedema to be admitted to the ICU. There is also a limitation due to NPL being performed by both ENT and ED providers, as variability in training and operator experience could lead to interobserver variability regarding the evaluation of structures. Our study is a single-center retrospective analysis which contributes to its limitations as well. Finally, 40 (29.9%) patients did not have an NPL performed, which may have had an impact on the significance of our findings, as patients who were intubated but did not have NPL may have had normal NPL findings. Such findings could dramatically change the results of this study. 

## Conclusions

Nasopharyngolaryngoscopy findings independent of precise anatomical location in patients with angioedema may be an important tool in determining the need for ICU admission and predicting the need for intubation. Although our study examined patients with angioedema admitted to the ICU only, there was a correlation between NPL findings and the need for intubation, and the presence of normal NPL findings had a negative predictive value of 86.5% for requiring intubation. Larger multi-center trials aimed at elucidating this relationship and the efficacy of NPL as a screening and triage tool for patients with angioedema are required.
